# Spatial patterns and epidemiological characterization of suicides in the Chapecó micro-region, Santa Catarina, Brazil: an ecological study, 1996-2018

**DOI:** 10.1590/S2237-96222023000100007

**Published:** 2023-04-14

**Authors:** Daniel Hideki Bando, Lucas Azeredo Rodrigues, Laura Lange Biesek, Deoclécio Luchini, Paulo Roberto Barbato, Graciela Soares Fonsêca, Jane Kelly Oliveira Friestino

**Affiliations:** 1 Universidade Federal de Alfenas, Instituto de Ciências da Natureza, Alfenas, MG, Brazil; 2 Universidade Federal de Santa Catarina, Florianópolis, SC, Brazil; 3 Universidade Federal da Fronteira Sul, Campus Chapecó, Chapecó, SC, Brazil

**Keywords:** Suicide, Mortality, Spatial Analysis, Space-Time Clustering, Epidemiological Monitoring, suicidio, Mortalidad, Análisis Espacial, Agrupamiento Espacio-Temporal, Monitoreo Epidemiológico, Suicídio, Mortalidade, Análise Espacial, Conglomerados Espaço-Temporais, Monitoramento Epidemiológico

## Abstract

**Objective:**

to identify spatial clusters of suicide and its epidemiological characteristics in the Chapecó (SC) micro-region from 1996 to 2018.

**Methods:**

this was an exploratory ecological study, using data from the Mortality Information System; specific suicide rates and relative risks (RR) were calculated with a 95% confidence interval (95%CI); the scan statistic was used for spatial analysis.

**Results:**

there were 1,034 suicides (13.7/100,000 inhabitants), with a male/female ratio of 3.79; the ≥ 60 age group was at higher risk for both sexes; a high risk cluster was found in the southwest region (RR = 1.57) and a low risk cluster in the southeast region, including Chapecó itself (RR = 0.68); risk of suicide among widowed (RR = 3.05; 95%CI 1.99;4.67), separated (RR = 2.48; 95%CI 1.44;4.27), and married (RR = 1.97; 95%CI 1.54;2.51) people was higher than among single people. The main methods were hanging (81.2%) and firearms (9.7%).

**Conclusion:**

there was a higher risk of suicide in the elderly, male and widowed people. Hanging was the most frequent method and risk clustering was found in the southwest.

## Resumo

**Objetivo:**

identificar conglomerados espaciais do suicídio e características epidemiológicas na microrregião de Chapecó, SC, Brasil, 1996-2018.

**Métodos:**

estudo ecológico exploratório, com dados do Sistema de Informações sobre Mortalidade; foram calculadas taxas específicas de suicídio, e riscos relativos (RR) com intervalo de confiança de 95% (IC_95%_); na análise espacial, utilizou-se estatística *scan*.

**Resultados:**

1.034 suicídios (13,7/100 mil habitantes), a razão sexo masculino/feminino de 3,79; a idade ≥ 60 anos apresentou maior risco para ambos os sexos; observou-se um conglomerado de alto risco na região sudoeste (RR = 1,57) e um de menor risco a sudeste, incluindo Chapecó (RR = 0,68); risco de suicídio entre viúvos (RR = 3,05; IC_95%_ 1,99;4,67), separados (RR = 2,48; IC_95%_ 1,44;4,27) e casados (RR = 1,97; IC_95%_ 1,54;2,51) maior que entre solteiros; principal método, enforcamento (81,2%), seguido por uso de arma de fogo (9,7%).

**Conclusão:**

maior risco de suicídio para idosos, sexo masculino e viúvos; enforcamento foi mais frequente; conglomerado de risco a sudoeste.

## Resumen

**Objetivo:**

identificar agregados espaciales de suicidio y el perfil epidemiológico en la microrregión de Chapecó (SC), 1996-2018.

**Métodos:**

estudio ecológico exploratorio, con datos del Sistema de Información de Mortalidad. Fueron calculadas tasas específicas de suicidio y riesgos relativos (RR) con intervalo de confianza 95% (IC95%). Para el análisis espacial se utilizó la estadística scan.

**Resultados:**

hubo 1.034 suicidios (13,7/100.000 habitantes), razón sexo masculino/femenino de 3,79. El grupo de ≥ 60 años presentó mayor riesgo para ambos sexos. Se observó un agregado de alto riesgo en la región suroeste (RR = 1,57) y un agregado de menor riesgo en el sureste (RR = 0,68). El riesgo de suicidio entre viudos (RR = 3.05; IC_95%_ 1,99;4,67), separados (RR = 2.48; IC_95%_ 1,44;4,27) y casados (RR = 1.97; IC_95%_ 1,54;2,51) fueron mayores que entre solteros. El principal medio fue el ahorcamiento (81,2%), seguido de las armas de fuego (9,7%).

**Conclusión:**

hubo mayor riesgo de suicidio en ancianos, sexo masculino y viudos. Ahorcamiento fue el método más frecuente y se observó agrupación de riesgos hacia el Suroeste.

**Table:** 

Study contributions	
**Main results**	A cluster of 19 municipalities at high risk for suicide was identified in the southwest of the Chapecó-SC micro-region. Risk of suicide was higher among males, elderly people and the widowed, mainly by hanging, followed by use of firearms.
**Implications for services**	Health services need to check service availability in the municipalities of that micro-region, especially in the high suicide risk area. Health professionals need to pay greater attention/care to the profile of male, the elderly, the widowed and the rural environment.
**Perspectives**	New studies with different methodological approaches (e.g. with details of the death certificate, psychological autopsies) should be conducted to clarify some points of the present study, such as the rural environment and suicide in the Southern region.

## INTRODUCTION

Suicide is a complex phenomenon and involves multiple biological, psychological, clinical, social and environmental factors, combined with experiences of loss and trauma.^1^ ^,^ ^2^ Death by suicide causes a great psychological and health impact on families, and extends to society as a whole.^3^ The World Health Organization (WHO) estimates that approximately 800,000 people lose their lives as a result of suicide every year, corresponding to a rate of 9.0/100,000 inhabitants, making this condition one of the 20 leading causes of death worldwide.^4^ Despite a global reduction of almost 10% in standardized suicide rates between 2010 and 2016, there was a 6% increase in these rates in the Americas.^4^ Suicide prevention actions are considered a priority for the WHO: one of the Sustainable Development Goals is to reduce the global suicide mortality rate by one third by 2030.^4^

In Brazil, although the suicide rate is below the global rate (6.4/100,000 inhab.),^4^ occurrence of suicide has tended to increase in recent years.^5^ The southern region of the country is known for high suicide rates above the national average.^6^ ^,^ ^7^ Traditions of European colonization, notably German, farming characteristics, low schooling and high incidence of mental disorders may contribute to this scenario.^6^ ^,^ ^8^ According to the 2017 Global Burden of Disease study estimate, Southern Brazilian also had the highest prevalence of mental disorders, with the state of Santa Catarina in the lead (15.5%), followed by Rio Grande do Sul (15.2%) and Paraná (15.0%).^9^ Considering Brazil’s different territories and their characteristics, important variations were found in suicide rates between states,^5^ municipalities^6^ and neighborhoods (of a municipality),^10^ possibly related to sociocultural factors.

In Santa Catarina, for example, the city of Chapecó plays a prominent role through its central regional location in relation to the southern border of Brazil (western Santa Catarina, northwestern Rio Grande do Sul and southwestern Paraná), with its predominant European colonization - mainly German, Italian and Azorean -, despite the socio-spatial formation founded on the occupation of land by aboriginal Indigenous communities and people of mixed race (White and Indigenous). The process of colonization, predominantly economic and ethnic-cultural, which began in the 20^th^ century, gradually brought new regional dynamics within the state, mainly through restructuring of production processes, and the reflection of this on the rural-urban relationship and the setting up of agro-industry networks, especially with effect from the mid-1980s.^11^ The advancement of production chains and increased demand for greater fluidity in circulation throughout the territory led to the creation of new networks, which explains the different socioeconomic contrasts between the resident populations, urban and rural, in the cities of the region. 

According to data from the Health Ministry Mortality Information System (Sistema de Informações sobre Mortalidade - SIM),^12^ between 1996 and 2018 the mortality rate due to external causes reported for the Chapecó micro-region was 69.12/100,000 inhab., slightly above the rate for Santa Catarina state as a whole (65.74/100,000). In the Chapecó micro-region, the main burden among external causes corresponds to transport accidents (41.9%), followed by homicides (19.0%), other external causes (18.9%) and suicide (16.6%). In the state as a whole, proportional mortality due to transport accidents was estimated to be 44.6%, followed by mortality due to “other external causes” (21.5%), “homicide” (17.3%) and “suicide” (12.8%). The Chapecó suicide rate therefore stands out a being above the average for Santa Catarina.

Identification of suicide clusters by municipality by means of spatial analysis proves to be an important strategy for the formulation of public prevention policies targeting the characteristics of the population at risk. The objective of this study was to identify spatial clusters of suicide and its epidemiological characteristics in the municipalities comprising the Chapecó micro-region in the state of Santa Catarina between 1996 and 2018.

## METHODS

We conducted an exploratory ecological study with spatial analysis of the suicide rates in the municipalities comprising the Chapecó micro-region in the state of Santa Catarina from 1996 to 2018. 

**Figure 1:**
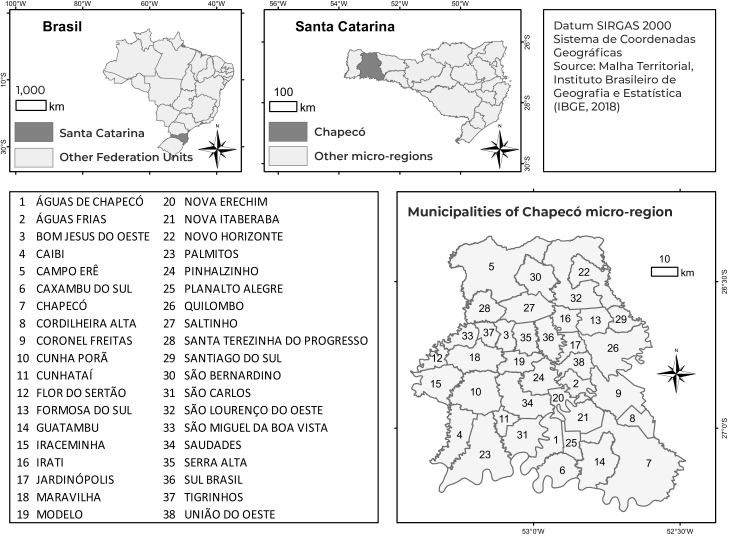
- Map showing location of the study area and describing the municipalities that comprise the Chapecó micro-region, Santa Catarina, Brazil

Santa Catarina forms part of the Southern region of Brazil and is comprised of 295 municipalities grouped into 20 micro-regions. The state covers an area of 95,730.684 km² and is the smallest in the Southern region. In 2020 it had an estimated population of 7,252,502 inhabitants.^13^ The Chapecó micro-region of is made up of 38 municipalities and in 2020 had an estimated population of 455,210 inhabitants^13^ (Figure 1). In 2019, the micro-region’s gross domestic product (GDP) was BRL 19.95 billion, the fifth largest GDP among the state’s 20 micro-regions.^13^ According to the most recent demographic census carried out by the Brazilian Institute of Geography and Statistics (Instituto Brasileiro de Geografia e Estatística - IBGE) in 2010, the proportion of low-income people (less than half a minimum wage per capita) in the Chapecó micro-region was 14.19%, slightly above the proportion for Santa Catarina as a whole (13.86%), coming in 12^th^ place in descending order in the state.^13^ The micro-region has two hospitals with psychiatric beds (32 beds in total), located in the municipalities of Palmitos and Quilombo. In addition, six municipalities have Psychosocial Care Centers (Centros de Atenção Psicossocial - CAPS I), which cover 16 municipalities, while only the municipality of Chapecó has specialized CAPS III Alcohol and Drugs services and CAPS services for children.

The clusters of suicide cases in the Chapecó micro-region, during the period assessed, were classified according to the following variables:

- sex (male; female);
- age (in completed years: 10-19; 20-39; 40-59; 60 and over);
- marital status (single; married; separated; widowed);
- suicide method (hanging; firearm; poisoning; other methods);
- place (municipality) of residence of the dead person; and
- year of death (between 1996 and 2018).

Mortality data were extracted from the Health Ministry Mortality Information System (Sistema de Informações sobre Mortalidade - SIM), via the Brazilian National Health System Department of Information Technology (Departamento de Informática do Sistema Único de Saúde - DATASUS),^12^ according to municipality of residence of the registered deaths. Deaths due to suicide correspond to the “X60 to X84 - intentional self-harm” codes contained in the Tenth Revision of the International Statistical Classification of Diseases and Related Health Problems (ICD-10). The initial year of the study was defined as 1996 because from then on the SIM adopted ICD-10 for classifying the diseases and health problems of the Brazilian population; and 2018 as the final year, precisely because it was the last year with these records available while the study was being conducted. Population data by sex, age group and marital status were obtained from the 2010 Census, along with the respective projections for the intercensal periods.^13^

The specific suicide mortality rate^14^ was calculated based on the number of deaths due to suicide of residents in a given location, taken as the numerator, and taking the total resident population as the denominator, multiplied by 100,000, in the period considered. For the intercensal periods we used the sum of the IBGE population projections for each year (also available on the DATASUS website).^12^ The cartographic basis of the study consisted of the territorial grid of the Chapecó micro-region, segmented by municipalities, in shapefile format.^13^

The spatial analysis used geoprocessing techniques to prepare thematic maps, according to the chorochromatic and choropleth cartographic methods,^6^ ^,^ ^10^ ^,^ ^15^ using the ArcGIS 10.6 geographic information system. Scan statistics were applied using the SaTScan application to identify high-risk spatial clusters and those at lower risk for suicide.^6^ ^,^ ^10^ ^,^ ^16^ This statistic inserts a circular window of variable size on the map surface, in such a way as to allow its center to move - for a given position and size - and the window-circle to include a different set of neighboring municipalities. If this “window’s” view reaches the geographic centroid of a neighboring municipality, the entire area of the municipality will be taken into consideration in the circumscribed cluster. The “age group” and “sex” variables contributed to this analysis as offset adjustment variables. Regarding the parameters, the maximum cluster size was defined as 50% of the population at risk. Under the null hypothesis, we checked whether clustering occurred by chance, using the likelihood ratio test based on Monte Carlo simulations. In the case of Chapecó we used a 5% significance level; that is, the null hypothesis was rejected when the p-value was < 0.05 for the most likely cluster, and for the secondary clusters.^16^

Regarding the epidemiological profile, suicide rates were calculated by sex, age group and marital status. For marital status, relative risks (RR) were calculated taking the group of single people as the reference group, with 95% confidence intervals (95%CI), and two periods of analysis: 1996 to 2004; and 2006 to 2014. It should be noted that the categorization of the population by marital status is only available for the years in which the demographic census was conducted, namely, 2000 and 2010. Therefore, two time frames were used, taking the population of the census years within the selected time periods, 1996-2004 and 2006-2014, considering four years before and four years after the census years. The calculation of proportional mortality according to the suicide method used, in the period 1996-2018, was defined based on four categories: poisoning; hanging; use of a firearm; and other methods.

The study used aggregated public domain data provided by DATASUS and, therefore, the study did not need to submitted for appraisal by a Research Ethics Committee.

## RESULTS

In the period from 1996 to 2018, there were 1,034 suicides in the study area, corresponding to 13.74/100,000 inhabitants: 21.64/100,000 males and 5.72/100,000 females. The male/female ratio was 3.79. The 10-19 year age group had the lowest rates for both sexes. An increase in suicide rates was found as age increased, being higher in the population aged 60 or over (Table 1). 

**Table 1:** - Number of suicide cases and suicide rates (per 100,000 inhabitants), by sex and age group, Chapecó micro-region, Santa Catarina, Brazil, 1996-2018

Age group (at last birthday)	Males			Females			Total		
	N	Population	Rate	N	Population	Rate	N	Population	Rate
10-19	37	822,147	4.5	22	792,356	2.8	59	1,614,503	3.7
20-39	269	1,506,011	17.9	73	1,478,703	4.9	342	2,984,714	11.5
40-59	330	1,026,198	32.2	80	1,020,899	7.8	410	2,047,097	20.0
≥ 60	182	427,503	42.6	41	497,317	8.2	223	924,820	24.1
Total	818	3,781,859	21.6	216	3,789,275	5.7	1,034	7,571,134	13.7

**Figure 2:**
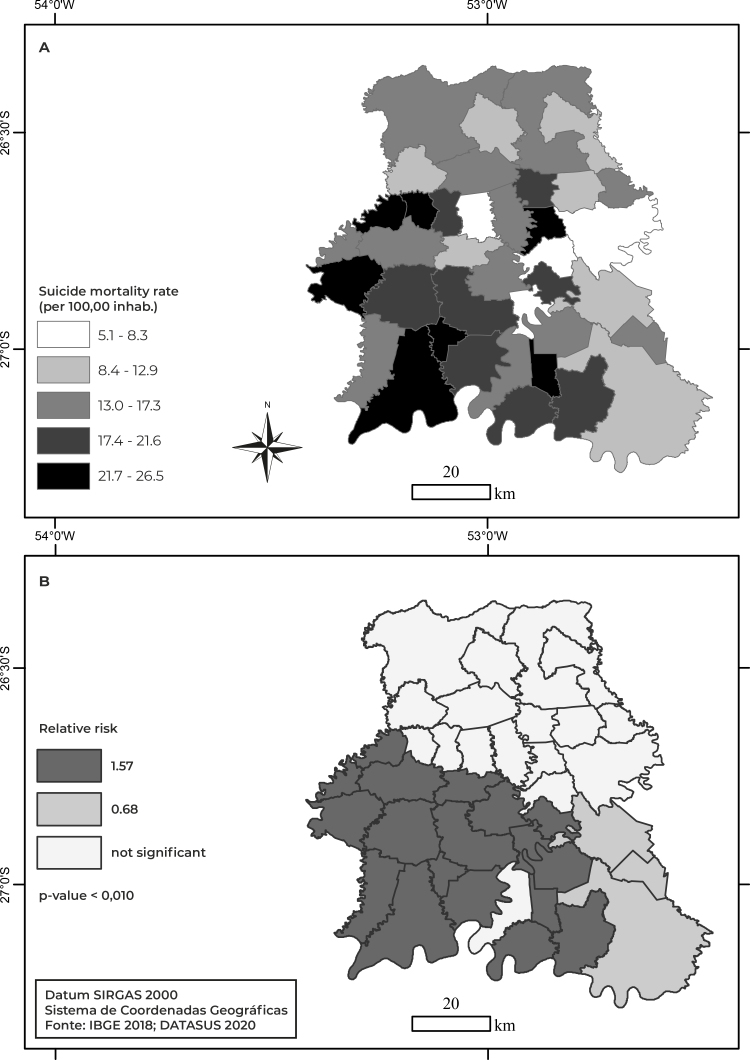
- Suicide rate distribution by municipality and spatial clusters with high risk and low risk of suicide, Chapecó micro-region, Santa Catarina, Brazil, 1996-2018

Municipalities in the west of the Chapecó micro-region, such as Tigrinhos, São Miguel da Boa Vista, and in the southwest, such as Palmitos and Iraceminha, had the highest suicide rates: above 21.70/100,000 inhab. Municipalities close to the center and in the east, such as Nova Erechim, União do Oeste, Quilombo and Serra Alta, had the lowest rates: up to 8.32/100,000 inhab. In the southeast of the Chapecó micro-region, in Coronel Freitas, suicide rates were below average, varying between 8.43 and 12.91/100,000 inhab. (Figure 2A). The spatial scanning test identified two significant spatial clusters: one of high risk, composed of 19 municipalities located in the southwest of the micro-region (RR = 1.57; p-value < 0.001), an area where the risk of suicide increased by 57%; and the lowest risk cluster, formed by three municipalities in the southeast of the micro-region, Cordilheira Alta, Coronel Freitas and Chapecó (RR = 0.68; p-value < 0.001), where risk of suicide was 32% lower (Figure 2B). 

Table 2 presents the suicide rates and respective RRs according to marital status. In both periods, 1996-2004 and 2006-2014, the group of single people had the lowest rate, and was used as the reference group. From 1996 to 2004, risk of suicide among married people was almost twice that of single people; in the case of separated people, risk was 2.48 times greater, and among widowed people it was more than 3 times greater than risk among single people. In the period from 2006 to 2014, risk of suicide among married people remained at a level similar to that of the previous period, while for widowed people, risk was 2.21 times greater, and for separated people it was 2.87 times greater than among single people.

**Table 2:** - Suicide rates (per 100,000 inhabitants) and relative risks, by marital status (n = 782), Chapecó micro-region, Santa Catarina, Brazil, 1996-2004 and 2006-2014

Marital status	1996-2004				2006-2014			
	N (%)	Population	Rate	Relative risk (95%CI^a^)	N (%)	Population	Rate	Relative risk (95%CI^a^)
Single	94 (25.3)	1,174,932	8.00	1.00	119 (29.0)	1,539,963	7.73	1.00
Married	200 (53.8)	1,271,538	15.73	1.97 (1.54;2.51)	198 (48.3)	1,317,357	15.03	1.95 (1.55;2.44)
Separated	15 (4.0)	75,753	19.80	2.48 (1.44;4.27)	33 (8.0)	148,608	22.21	2.87 (1.95;4.23)
Widowed	27 (7.3)	110,790	24.37	3.05 (1.99;4.67)	26 (6.3)	152,118	17.09	2.21 (1.45;3.38)
Unknown	36 (9.7)	-	-		34 (8.3)	-	-	
Total	372 (100.0)				410 (100.0)			

a) 95%CI: 95% confidence interval.

**Figure 3:**
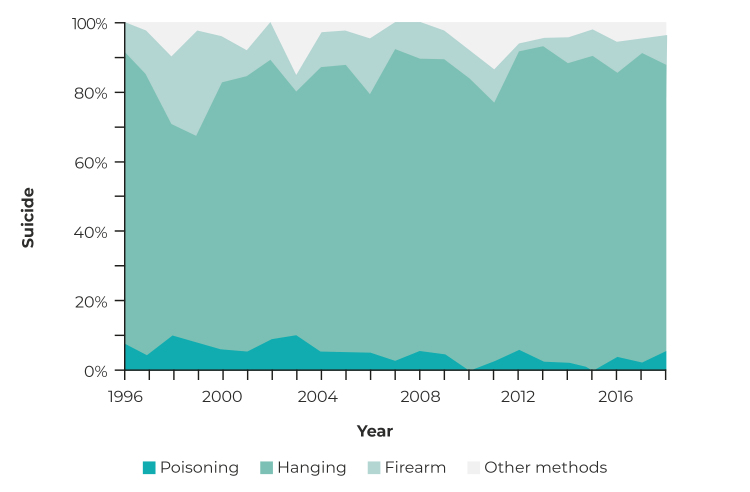
- Proportion of mortality per suicide method (n = 1,034), Chapecó micro-region, Santa Catarina, Brazil, 1996-2018

As for the suicide method used, most occurred by hanging (81.2%), followed by the use of a firearm (9.7%) and poisoning (4.5%) (Figure 3). Use of a firearm accounted for 30.0% of the means used to commit suicide in 1999; there was a decrease in this type of suicide in the subsequent period, reaching values below 9.1% from 2009 onwards. Suicide by poisoning showed two peaks in the proportion of its occurrence: 9.8% in 1998 and 2003, while from 2004 onward it was always less than 6.1%.

## DISCUSSION

Two spatial clusters were found in the Chapecó micro-region, one with high risk and the other with lower risk of occurrence of suicide. The highest risk group consisted of male aged 60 years or older. Risk of suicide among widowed, separated and married individuals was higher when compared to single individuals. Hanging was the most used method, followed by use of a firearm. 

In agreement with the present study, a descriptive investigation, carried out in Brazil for the period 2000 to 2012, also identified the elderly as the highest risk group,^17^ corroborating global evidence showing that the risk of suicide was up to 8 times higher in this age group compared to other age groups.^18^ Suicide among the elderly is a public health problem in several countries, such as the United States, where mortality rate among elderly White people due to this cause has reached 48.7/100,000 inhab. Suicide rates increase with advancing age, while suicide attempts decrease, as does the prevalence of psychiatric treatment. Suicide in the elderly is related to recognized factors, such as social isolation, hidden grief, presence of chronic diseases, dementia, mental disorders, disabilities; and adverse situations, such as being retired.^19^

People with suicidal behavior tend to have less social support, less sense of belonging and greater difficulty in interpersonal relationships. Social support effectively provides a protective effect against common stressors of advanced age.^19^ Considering that the population is rapidly becoming older in the Chapecó micro-region^13^ and the higher risk of suicide among the elderly, this is a factor that deserves attention through public policies. Health services, such as Primary Care and actions aimed at mental health, especially prevention programs, could monitor and provide greater attention to groups identified as being more vulnerable, such as those identified in this research: male, elderly and widowed people.

In Brazil, an ecological study using scan statistics detected spatial clusters among the municipalities analyzed for the period from 1990 to 2015, identifying suicide risk areas in the Southern region of the country; as well as determinants such as sociocultural, economic and psychobiological aspects of gaucho farmers, especially, in addition to the demanding standards of social behavior required by German immigrant traditions.^6^ These factors suggest a relationship with the spatial cluster of high suicide risk identified in the present study: a large part of the population of the municipalities located in the southwest of the Chapecó micro-region are descendents of European immigrants (Germans, Italians, Poles), coming originally from the state of Rio Grande do Sul and who continue undertaking similar economic activities.^20^

Moreover, the rural environment is associated with a cultural identity of honorableness, family aspects and gender attributions. Generations of suicidal behavior, as a way of responding to dilemmas and suffering, must be considered, ^21^ given that family history of suicide is a known risk factor, both from a biological and psychological point of view.^1^ Data on the suicide rate in Germany (13.6/100,000 inhab.) corroborate this assumption, as it is close to the rate found in the Chapecó micro-region.^18^ In São Paulo City, a study with individual data also identified greater risk of suicide among European immigrants - a rate of 12.1/100,000 inhab. and RR = 2.78 -, compared to native people.^22^

The proportion of the rural population was found to be high in municipalities falling into the high suicide risk cluster, such as Flor do Sertão, São Miguel da Boa Vista, Cunhataí and Iraceminha, while in Nova Itaberaba, Guatambu, Planalto Alegre, Águas Frias and Caxambu do Sul, which also fell into the high risk cluster, the rural population accounted for a smaller proportion. Despite the evident disagreement in the findings, we also found that in the three municipalities of the cluster with the lowest suicide risk, Cordilheira Alta, Coronel Freitas and Chapecó, the percentage of people living in the rural area was lower.^13^ This information, in particular, suggests a possible association between living in rural areas and risk of suicide, in agreement with the conclusions of a systematic review using meta-analysis of 53 cohort studies, case-control studies and cross-sectional studies, published between 2006 and 2017 on rurality and suicide in English-speaking and high-income countries, which identified the rural environment as a risk factor for suicide, mainly among men.^23^ In Brazil, a regional ecological study covering the period from 1998 to 2002, showed that degree of rurality was directly related to suicide.^24^ The rural environment may be associated with greater difficulty in accessing the health system, poorer living and working conditions, social stigma directed towards mental health and seeking help, as well as greater access to firearms.^23^ ^,^ ^24^ In Brazil as a whole, inequality between urban and rural areas with regard to access to health services is a recognized fact, and access is even lower among elderly people living in the countryside.^25^ Family farming is a striking reality in western Santa Catarina.^20^ Another important factor related to the rural environment is the presence of pesticides, whereby inappropriate use, without appropriate personal protective equipment, ease of access to them and their being lethal suggest an association between depression symptoms and suicide among the population residing in these areas.^1^ ^,^ ^26^ Between 2000 and 2012, 40% of suicides due to poisoning in Brazil resulted from use of pesticides.^17^ Furthermore, being physically disabled can increase the risk of suicidal ideation. One third of accidents in the rural environment result in permanent harm and are underreported, which may, in the future, serve as a trigger for suicide.^27^

Widowhood can also be a stressful event associated with suicide.^21^ We identified greater relative risks among widowed, separated and married people in the period defined for the present study. Additionally, according to the 2010 Census, of the 19 municipalities that make up the high suicide risk cluster, the percentage of widowed people is above the average for the state of Santa Catarina (4.8%)^13^ in 15 of them. In the state of Rio Grande do Sul, between 1980 and 1999, the suicide rate was higher for widowed people, followed by married, single and separated people.^21^ On the other hand, an observational study conducted in the coal mining region of Santa Catarina (the same state as in our study), based on data on suicides between 1980 and 2007, identified higher risk of occurrence among married couples.^28^ These results from the Southern region of Brazil differ from the literature, according to which being married is a protective factor against suicide. In São Paulo City, taking married couples as a reference, higher risk of suicide was found among single, divorced and widowed individuals.^22^ A systematic review which performed a meta-analysis of cohort studies published between 1994 and 2007, on marital status and suicide in the elderly, also found that married individuals show protection against suicide, compared to unmarried individuals, i.e. widowed, divorced, separated and single people.^29^

The most used suicide methods in the Chapecó micro-region were hanging (81.2%), use of firearms and poisoning. These findings corroborate those of another study carried out in Santa Catarina, which found that hanging was the main method for both sexes, followed by use of firearms and pesticide poisoning among males; and medication and drug poisoning and use of firearms among women.^30^ In Rio Grande do Sul, likewise, hanging was the most used method, accounting for 72.5% of occurrences.^7^ In Brazil, between 1996 and 2016, the most used methods were hanging (58%), use of firearms (15%) and self-poisoning with pesticides or other chemicals (10%). Hanging has shown an increasing trend in Brazil: in 2016, 72% of male suicides and 56% of female suicides were due to hanging.^30^ Greater use of hanging can be attributed to the availability of materials and the perception that this method leads to a quick, clean and painless death. Suicide by hanging was higher in elderly men, in the rural environment and in people with a low level of schooling.^30^ Knowledge of the methods used to commit suicide is important for implementing prevention strategies. At the same time, it is a challenge, as in the case of hanging, this commonly happens at home. Borge-Santos & Wang suggest an in-depth discussion of the topic among health professionals, family members and health service managers working on the front line of mental health care, as well as research on the perception and meaning of suicide by hanging in different cultures.^30^

The present study has some limitations. The first is inherent to its design, and may incur an ecological fallacy, that is, attributing to individuals associations that were found in the general population. Possible misclassifications recorded at the time of death also reduce confidence in the findings. Procedures without standardization, as well as cultural and social values, can impact death records, leading to underreporting or incorrect classification of suicide, such as religious issues, stigmas or even legal issues, such as loss of life insurance.^10^ This type of bias is inevitable in ecological studies on suicide that use the Mortality Information System database.^12^ There are exceptions, as in the case of São Paulo City where the Mortality Information Improvement Program (Programa de Aprimoramento das Informações de Mortalidade - PRO-AIM) was created in 1989, aiming to develop methods for obtaining and improving information on the Death Certificates of all suicide cases occurring in the city.^22^ New studies, adopting different methodologies, such as, for example, a qualitative approach through interviews with health professionals and family members of people who committed suicide (psychological autopsies), can help to understand the cause of the phenomenon and assess its impact on families and society,^3^ providing a greater range of resources for those involved in dealing with these situations.

In conclusion, this study identified a high suicide risk cluster, formed by 19 municipalities in the southwest of the Chapecó micro-region, in the state of Santa Catarina, worthy of special public health attention. Males, the elderly and widowed people were at greater risk of suicide, having hanging as the main method, followed by use of a firearm. The results of this study also suggest greater risk of suicide in rural environments. New research may further identify the population groups/segments most vulnerable to suicide, providing input for health services for formulating public policies aimed at suicide prevention and postvention.
